# Fibro-adipose vascular anomaly (FAVA) - diagnosis, staging and management

**DOI:** 10.1186/s13023-023-02961-6

**Published:** 2023-11-07

**Authors:** Huaijie Wang, Chong Xie, Weilong Lin, Peihua Wang, Weijia Yang, Zhengtuan Guo

**Affiliations:** Department of Pediatric Surgery& Vascular Anomalies, Xi’an International Medical Center Hospital, Xi’an, 710100 China

**Keywords:** Fibro-adipose vascular anomaly, PROS, Venous malformation, Surgery, Endoscopic, Sclerotherapy, Sirolimus, Alpelisib

## Abstract

**Background:**

The diagnosis and treatment of fibro-adipose vascular anomaly (FAVA) of the limb remains challenging since this entity is rare and complex. This paper is aimed to describe the clinical and imaging features, staging and management of this underrecognized disease of the limb.

**Material and method:**

Patients diagnosed with FAVA and managed between September 2019 and May 2022 in department of pediatric surgery & vascular anomalies of Xi’an international medical center hospital were retrospectively reviewed. Data extracted include age at presentation, previous diagnosis, affected muscles, symptoms, previous treatment, our management, and follow-up.

**Results:**

Thirty-two patients with FAVA were diagnosed and managed in our center. There was a female sex predominance, with 23 female (72%) and 9 male (28%) in the cohort. Only one lesion was noticed during infancy; the remaining presented at age 1 to 20 years (median, 7 years). The most commonly involved muscles were gastrocnemius (14/32, 44%) and soleus (13/32, 40%). Swelling (mass), pain and contractures were the most common presentations. MRI featured a heterogeneous and ill-defined intramuscular high signal intensity. Diseases were staged according to clinical features: stage I (pain stage, n = 4), stage II (contracture stage, n = 20) and stage III (deformity stage, n = 8). Patients with stage I disease underwent radical resection and obtained a cure. Patients with stage II disease received radical resection and possible Achilles lengthening, having an outcome of cure. Personalized treatment was required in patients with stage III disease, including radical/partial/staged resection, Achilles lengthening/tenotomy, joint capsulotomy, neurolysis/neurectomy, tendon transfer, stretching exercises, and oral sirolimus/alpelisib. Significant improvement of symptoms was achieved in most.

**Conclusion:**

The most distinct features of FAVA include enlarging mass, severe pain and contracture. Based on distinct clinical and radiologic features, it is not difficult to make the diagnosis of FAVA. Earlier awareness of this disease can reduce misdiagnoses. Surgery-based comprehensive management can typically improve pain and contracture. Oral sirolimus or alpelisib plays an important role in treatment of unresectable lesions and major nerve involvement. Surgery alone can be curative in early stage FAVA.

## Introduction

Fibro-adipose vascular anomaly (FAVA) is a rare, complex mesenchymal entity with fibro-adipose venous infiltration of muscles and fascia [1–3]. This condition is typically featured by localized swelling, severe and persistent pain, phlebectasia, contracture, and joint deformity of the affected limb [1–3]. Misdiagnosis of this specific entity is frequent owing to the underrecognization, rarity, variable and overlapping symptoms, and confusing nomenclature of this condition [1–4].

Sclerotherapy is ineffective for improvement in symptoms of FAVA [5]. In literature, sirolimus has shown a rapid and promising outcome that resulted in relief of pain, regression of lesion, and improvement of quality of life in FAVA patients [6, 7]. Image-guided percutaneous cryoablation was also successfully used to control symptoms in FAVA at short-term follow-up [8]. However, radical ablation is difficult to achieve with these treatments. Some authors reported contracture subsequently increased and pain recurred in some patients after cryoablation [5]. Some reports showed surgery could potentially cure most FAVA patients [2, 3]. Results of our previous reports also suggested that surgery-based management was viable in FAVA patients [9, 10], where symptom improvement after successful treatment may be profound, including relief of pain and joint contracture. In current literature, treatment algorithm has not been established for FAVA. Here we present a cohort of FAVA patients and describe the clinical features, staging and management.

## Material and method

This retrospective study was approved by the Institutional Ethics Review Board of Xi’an International Medical Center. Data was extracted from the database between September 2019 and May 2022 at our Vascular Anomalies Center, including age at presentation, previous diagnosis, affected muscles, symptoms, previous treatment, our management, and follow-up.

The diagnosis of FAVA was based on following criteria [2, 3]: (1) A solid mass with persistent and severe pain and/or progressive contracture in child and youth; (2) Ultrasonographic finding of a solid, heterogeneous echogenic changes with phlebectasia entirely replacing the normal fibrillary pattern of muscle; Magnetic resonance imaging (MRI) demonstrating intramuscular heterogeneous high-signal infiltrating lesion with phlebectasia; and (3) Grossly and microscopically, significantly increased venous component, and fibro-adipose tissue replacing and infiltrating into muscle.

If the diagnosis of FAVA was established, diseases were staged according to clinical features: stage I (pain stage) - A local mass with pain, without significant contracture; stage II (contracture stage) - Significant contracture with limited joint mobility; stage III (deformity stage) - Joint deformity with ankylosis and possible limb length discrepancy (Table 1).

**Table 1:** Staging of FAVA

Stage	Clinical presentation	Treatment principle
I (Pain stage)	A mass with persistent and severe pain, without significant contracture	Radical resection
II (Contracture stage)	A mass with pain; Significant contracture with limited joint mobility	Radical resection Possible tendon lengthening and/or transfer Possible neurolysis Stretching exercises
III (Deformity stage)	A mass with pain; Joint deformity with ankylosis and possible limb length discrepancy	Radical resection if possible Tendon lengthening and/or transfer, tenotomy Neurolysis Joint capsulotomies Oral sirolimus/alpelisib Stretching exercises Possible osteotomy lengthening

The surgery for FAVA shared similarities with resection of other locally aggressive tumors. The surgery goal was for radical resection while restoring functions. Radical excision while preserving vital structures was a general principle. For stage I, wide local excision was preferred to minimize residual disease and/or recurrence. The extent of resection was determined according to MRI evaluation. Personalized management with preserving vital joint function was a general principle for patients with significant contracture/deformity. The overall goal of management was for pain relief, contracture relaxation, improvement of the mass effect, improved appearance and retained functional motion. Intraoperatively, the risk of recurrence/residual disease must be balanced against the function loss resulting from sacrificed critical muscles. All resections included excision of the affected muscle, thorough fasciotomy of all affected compartment, neurolysis of involved vital nerves, and capsulotomies of involved joints. Additional oral sirolimus/alpelisib, tendon lengthening and/or transfer, tenotomy, or stretching exercises was performed in selected patients. If the lesion was unresectable, oral sirolimus/alpelisib was recommended.

Pain and joint mobility were evaluated during planned follow-up.

## Results

### Previous diagnosis

Only one patient was correctly diagnosed with FAVA at referral. The most common previous diagnoses were venous malformation (VM) (n = 22), hemangioma (n = 5), arteriovenous malformation (n = 2), and growing pain (n = 2) (Table 2).

**Table 2:** Clinical characteristics of FAVA cohort

No	Gender	Age At Onset (y)	Age At Referral (y)	Symptoms	Locations	Affected Muscles	Interventions Before Referral	Stage	Interventions At Authors’ Institution	Clinical Change After Intervention(s)
1	M	1	11	Mass and severe pain Loss of ankle plantar flexion Muscle atrophy	Right leg and foot	Extensor digitorum longus Extensor hallucis longus	Sclerotherapy	III	Radical resection	No pain Loss of ankle plantar flexion Improving atrophy
2	F	3	11	Mass and severe pain Limited ankle dorsiflexion Muscle atrophy	Left leg	Soleus	Partial resection Sclerotherapy	II	Radical resection Stretching exercises	No pain Near normal range of ankle motion Improving atrophy
3	F	7	13	Mass and severe pain Limited ankle dorsiflexion Muscle atrophy	Right leg	Lateral gastrocnemius Soleus	Sclerotherapy Endovascular embolization	II	Radical resection	No pain Near normal range of ankle motion Improving atrophy
4	F	1	9	Mass and severe pain Contracture of knee Muscle atrophy	Left thigh	Biceps femoris Vastus lateralis Vastus intermedius	Sclerotherapy Oral sirolimus	III	Radical resection Relaxation of the knee Stretching exercises	No pain Near normal range of knee motion Re-walking Improving atrophy
5	F	2	9	Mass and severe pain Loss of ankle dorsiflexion Muscle atrophy	Left leg	Tibialis posterior Flexor hallucis longus Flexor digitorum longus Soleus	Sclerotherapy Oral sirolimus	II	Radical resection Achilles tendon lengthening Relaxation of ankle capsule Stretching exercises	No pain Normal range of ankle motion Improving atrophy
6	F	3	15	Mass and severe pain Loss of ankle dorsiflexion Pes cavus Contracture of knee Muscle atrophy	Right thigh, leg and foot	Soleus Gastrocnemius Biceps femoris Flexor digitorum longus Flexor digitorum brevis	Sclerotherapy Oral sirolimus	III	Staged resection Oral alpelisib	Mild pain Increased range of ankle motion Improving atrophy
7	M	8	9	Mass and pain	Left leg	Fascia muscularis	None	I	Radical resection	Normal
8	F	15	17	Mass and severe pain Limited forearm pronation	Left forearm	Supinator	Sclerotherapy Oral sirolimus Partial resection	II	Radical resection	No pain Loss of elbow motion after sclerotherapy
9	F	3 mo	1	Mass and severe pain	Left leg	Medial gastrocnemius Soleus	None	I	Radical resection	Normal
10	M	12	18	Mass and pain Contracture of wrist	Right forearm	Extensor carpi ulnaris	Sclerotherapy	III	Radical resection	Normal
11	F	5	10	Mass and severe pain Limited plantar flexion of ankle	Left leg	Tibialis anterior Extensor digitorum longus	Sclerotherapy Oral sirolimus	II	Radical resection Tendon transfer	Normal
12	M	8	13	Mass and severe pain Limited ankle dorsiflexion Muscle atrophy	Right leg	Soleus	Sclerotherapy Oral sirolimus	II	Radical resection Achilles tendon lengthening	No pain Near normal range of ankle motion Improving atrophy
13	F	7	14	Mass and severe pain Limited ankle dorsiflexion Muscle atrophy	Right leg	Lateral gastrocnemius	Sclerotherapy Endovascular embolization	II	Radical resection Achilles tendon lengthening	No pain Near normal range of ankle motion Improving atrophy
14	F	9	9	Mass and severe pain Limited ankle dorsiflexion	Left leg	Lateral gastrocnemius	Sclerotherapy	II	Radical resection	Normal
15	M	18	23	Mass and severe pain	Right thigh	Rectus femoris	Sclerotherapy	I	Radical resection	Normal
16	F	17	18	Mass and severe pain Limited plantar flexion of ankle	Right leg	Extensor digitorum longus	Sclerotherapy	II	Radical resection	Normal
17	F	9	10	Mass and severe pain Limited ankle dorsiflexion	Right leg	Medial gastrocnemius	Sclerotherapy	II	Radical resection (endoscopic)	Normal
18	M	9	11	Mass and severe pain Limited ankle dorsiflexion Muscle atrophy	Left leg	Medial gastrocnemius	Sclerotherapy	II	Radical resection Achilles tendon lengthening (endoscopic)	No pain Near normal range of ankle motion Improving atrophy
19	F	11	20	Mass and severe pain Limited ankle dorsiflexion	Left leg	Soleus	Sclerotherapy	II	Radical resection	Normal
20	F	2	14	Mass and severe pain Loss of ankle dorsiflexion Pes cavus Muscle atrophy	Right leg	Gastrocnemius Soleus	Sclerotherapy	III	Radical resection Achilles tenotomy Relaxation of ankle capsule Stretching exercises	No pain Loss of ankle plantar flexion Re-walking Improving atrophy
21	F	5	11	Mass and severe pain	Left thigh	Vastus medialis	Sclerotherapy	II	Radical resection	Normal
22	F	17	22	Mass and severe pain	Left thigh	Adductor magnus	Sclerotherapy	II	Radical resection	Normal
23	M	20	33	Mass and severe pain	Right thigh	Semimembranosus Semitendinosus	Sclerotherapy	II	Radical resection	Normal
24	F	10	11	Mass and severe pain Limited ankle dorsiflexion	Left leg	Soleus	None	II	Radical resection	Normal
25	F	9	11	Mass and severe pain Contracture of knee Limited ankle dorsiflexion Muscle atrophy	Right thigh and leg	Gracilis Saphenous fascia of leg Soleus	Sclerotherapy	II	Radical resection Stretching exercises	No pain Normal range of knee motion Near normal range of ankle motion Improving atrophy
26	F	6	11	Mass and severe pain Limited ankle dorsiflexion	Left leg	Lateral gastrocnemius	None	II	Radical resection (endoscopic)	No pain Near normal range of ankle motion
27	M	7	11	Mass and severe pain Limited ankle dorsiflexion Muscle atrophy	Left leg	Lateral gastrocnemius	Sclerotherapy	II	Radical resection (endoscopic)	No pain Near normal range of ankle motion
28	F	1	4	Mass and severe pain Limited ankle dorsiflexion Muscle atrophy	Left leg	Gastrocnemius	Sclerotherapy	II	Radical resection Achilles tendon lengthening Stretching exercises	No pain Near normal range of ankle motion Improving atrophy
29	M	3	10	Mass and severe pain	Right leg	Peronaeus longus	Sclerotherapy	I	Radical resection	Normal
30	F	5	23	Mass and severe pain Loss of ankle dorsiflexion Pes cavus Muscle atrophy	Right leg	Posterior muscle group	Sclerotherapy	III	Oral sirolimus Stretching exercises	Mild pain No change of the range of ankle motion Improving atrophy
31	F	5	13	Mass and severe pain Contracture of knee Loss of ankle dorsiflexion Muscle atrophy	Right thigh and leg	Biceps femoris Posterior muscle group of leg	Sclerotherapy	III	Oral sirolimus Stretching exercises	Mild pain Increased range of knee motion No change of the range of ankle motion
32	F	8	16	Mass and severe pain Loss of ankle dorsiflexion Pes cavus Muscle atrophy	Right leg	Posterior muscle group	Sclerotherapy Partial resection	III	Oral sirolimus Stretching exercises	Mild pain No change of the range of ankle motion Improving atrophy

### Clinical characteristics

There was a female predominance, with 23 female (72%) and 9 male (28%) in the cohort. Only one lesion was noticed during infancy; the remaining presented at age 1 to 20 years (median, 7 years). The most commonly involved muscles were gastrocnemius (14/32, 44%) and soleus (13/32, 40%). Two lesions involved the upper limb. Two lesions of the leg extended into foot. Two patients had two isolated lesions involved both thigh and leg. One patient had three isolated lesions involved thigh, leg and foot. No lesions involved two limbs (Table 2).

**Fig. 1:**
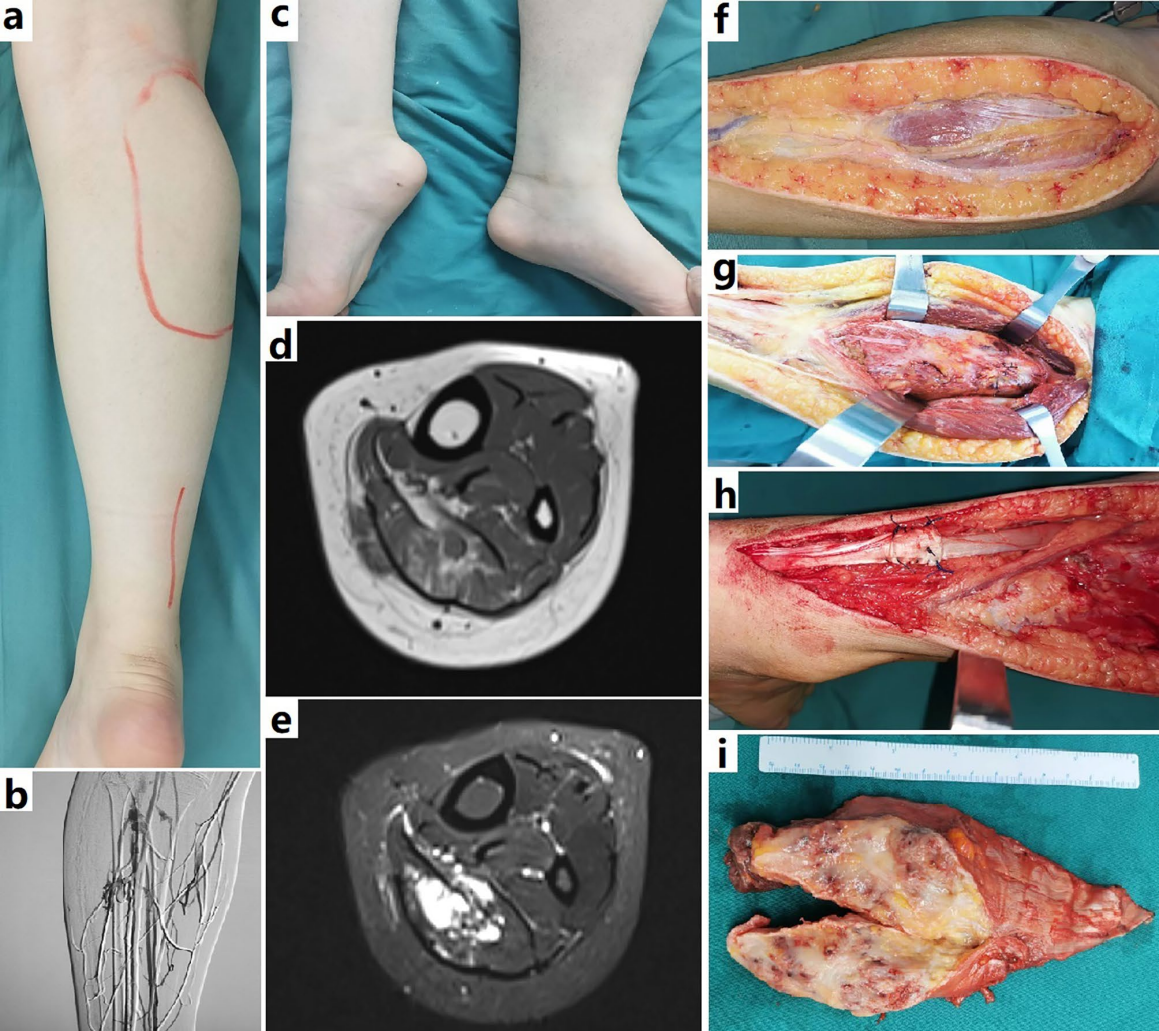
Surgical resection of a FAVA in calf. **a-c**, A girl was presented with Achilles contracture and a local mass in calf. Venography demonstrated anomalous-dilated veins, and normal orthotopic veins in the affected area. **d** and **e**, The soleus was infiltrated by diffuse heterogeneous high signal on MRI. The gastrocnemius atrophy was noted. **f**, The deep fascia over gastrocnemius was not obviously involved. **g**. The soleus underlying gastrocnemius was infiltrated by dense fibroadipose tissue and tufted venous component. **h**. Achilles lengthening was performed following radical resection of lesion because of significant contracture. **i**. Surgical specimen showed an intramuscular mass with unusually rounded venous nodules intermingled with dense fibrotic tissue and yellow fat

Usually, children presented with an enlarging painful mass, local swelling, and progressive limited range of motion (Fig. 1). The mass was typically hard on palpation. Pain was aggravated by exercise, and mildly mitigate by elevation of the affected limb. But the lesions didn’t shrink by elevating the limb on palpation. If limb atrophy and contracture were not present on initial presentation, they slowly developed within years. No lesion shrank with direct puncture and sclerotherapy, or trans-arterial embolization. Patients who had been treated with compression garments reported mild improvement of pain.

**Fig. 2:**
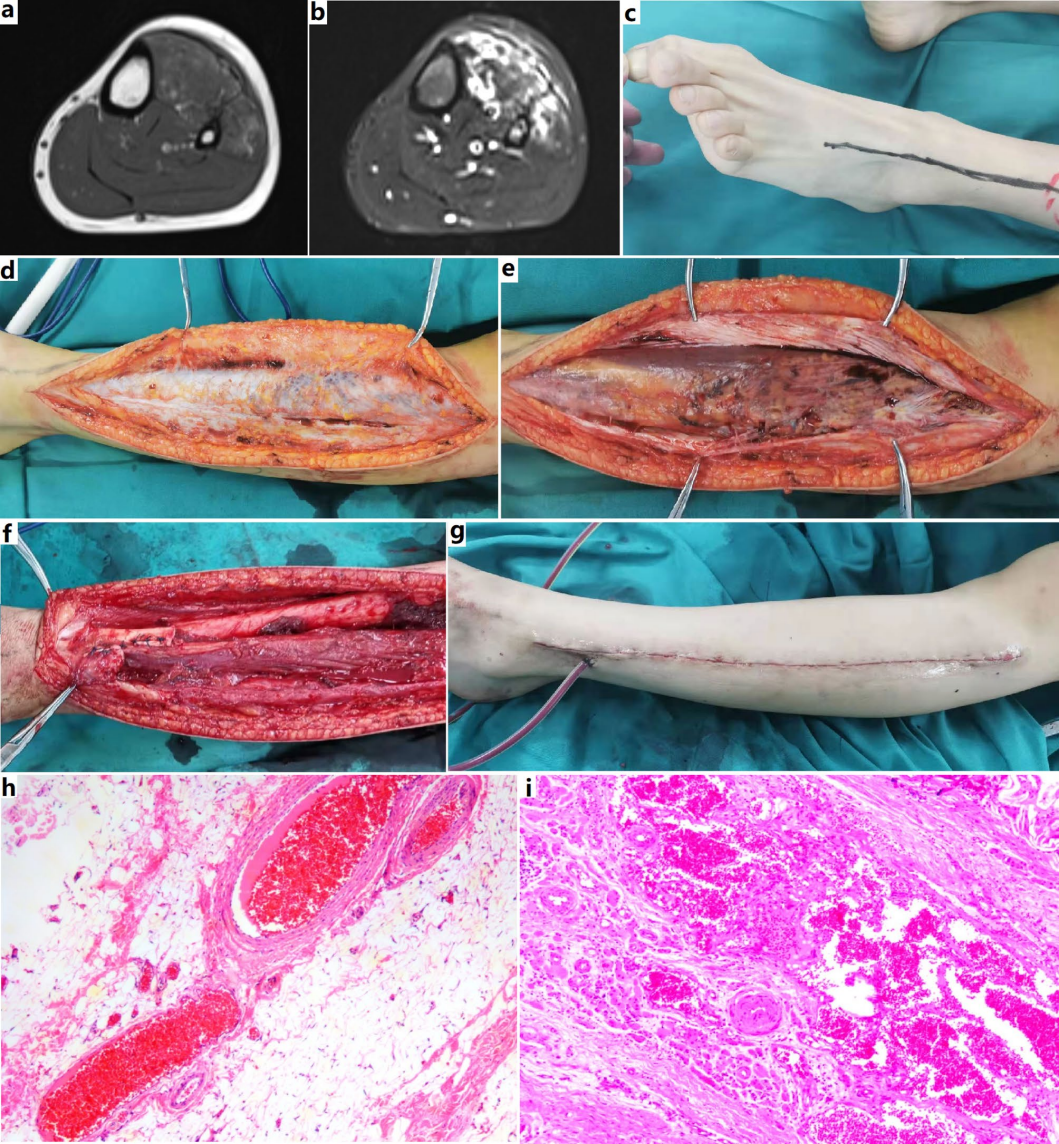
MRI and surgical resection of a FAVA in calf. **a**, Axial T1-weighted MRI. Both extensor longus digitorum and tibialis anterior muscle were diffusely infiltrated by heterogeneous mass with high fatty signal and low signal. **b**, Axial fat-saturated T2-weighted MRI revealed the lesion containing high fluid signal, the saturated fatty signal and other low signal. Note displaced and atrophied extensor hallucis longus, musculi peronaeus longus, and musculi peronaeus brevis, and the involved and thickened muscular fascia. **c**, Physical examination revealed no limitation of plantar flexion of the hallux, and limited plantar flexion of the second - fifth toes, suggesting extensor longus digitorum involvement. **d**, Operative findings. The deep fascia of extensor longus digitorum became thickened by fatty tissue and tufted frogspawn-like venous lakes infiltration. **e**, Dense, scarred fibroadipose tissue and venous anomaly infiltrated extensor longus digitorum and replaced the normal muscular pattern. **f**, After extensor longus digitorum and tibialis anterior muscle resection, tendon transfer was performed to reconstruct the dorsiflexion funtion of ankle. **g**, The appearance of sutured incision after placement of a vacuum drainage tube. **h** and **i** (HE staining, ×100), Histopathologic examination demonstrated excessive fat and fibrous tissue with abnormal medium-sized veins in deep fascia. Anomalous venous channels, fibrous and adipose tissue infiltrated the skeleton muscle and fascia. Abundant adipose and venous clusters, abnormal small-sized and medium-sized veins, and dense fibrous tissue were demonstrated, the latter concentrating around venous spaces. Thick-walled muscularized vasculars can be observed focally

Flexion contractures of the ankle and knee did not improve with use of an orthosis. The pain was too severe to accomplish the planned use of an orthosis or stretching exercises. Fourteen lesions extended across the intermuscular fascia to involve adjacent muscular in the same compartment (Fig. 2). No lesions extended across the interosseous membrane to involve another compartment. Two lesions only involved deep fascia. Twenty-eight lesions were confined to a single muscle and muscle group.

### Imaging finding

**Fig. 3:**
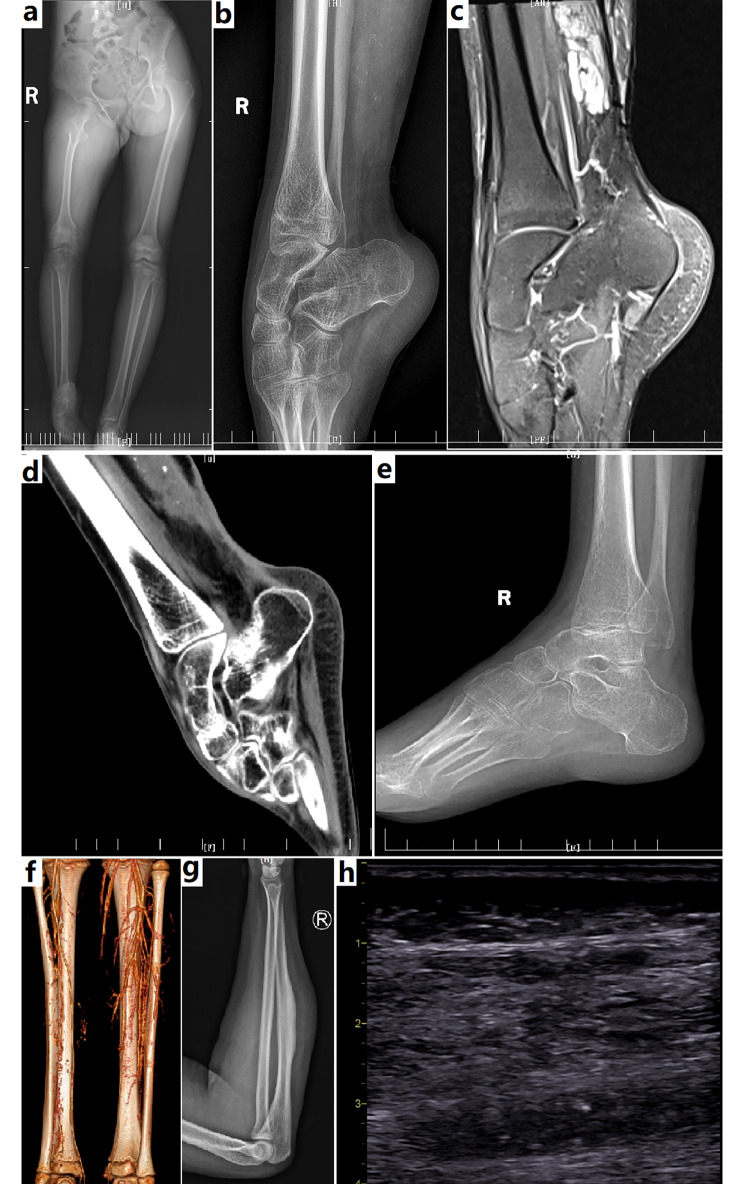
Imaging findings of FAVA. In a patient with FAVA of calf, **a**, X-ray plain radiography demonstrated pelvic tilt, leg length discrepancy, and fibular deformity. **b**, Ankle deformity with ankylosis, and pes cavus was noted on X-ray plain radiography. Intralesional foci of calcification or metaplastic bone tissue could also be seen. **c**, MRI revealed a mass with heterogeneous high signal infiltrating the posterior muscles in calf. **d**, CT scan demonstrated the mass contained infiltrative low density tissue, indicating a fatty component. **e**, She re-walked following radical resection, Achilles tenotomy, and relaxation of ankle capsule. **f**, In a case of FAVA in calf, contrast-enhanced CT scan revealed decreased arterial branches, and ectatic veins in the affected limb. Note the fibular deformity and leg atrophy. **g**, X-ray plain radiography revealed thickened cortex of the ulna in a case with FAVA of cubitalis posterior. **h**, FAVA manifested as a solid, noncompressible, and heterogeneous echogenic mass entirely replacing the normal fibrillary pattern within the affected muscle on ultrasonography study

**Fig. 4:**
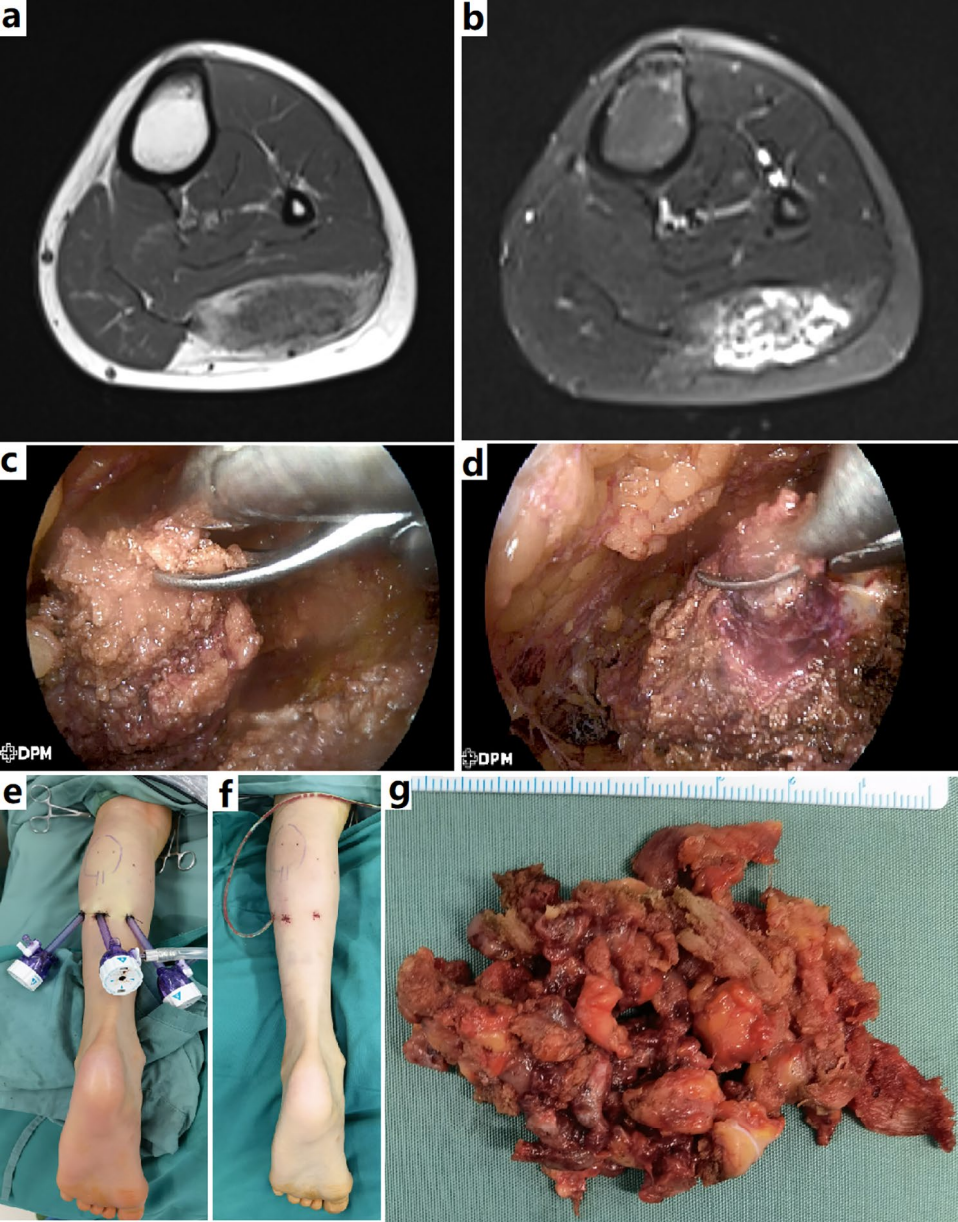
Endoscopic resection of a FAVA in calf. **a** and **b**, MRI demonstrated the lesion with typical FAVA signals infiltrated the lateral gastrocnemius. **c**, Endoscopic resection of the thickened deep fascia. **d**, Resection of the lateral gastrocnemius. The lesion had a typical appearance of FAVA. **e** and **f**, Port placement and postoperative appearance. **g**, Surgical specimen showed the lesion consisted of dense fibrous, yellow fatty and venous component

MRI studies were routinely performed without contrast. Images typically demonstrated a soft tissue lesion permeating the muscle and/or deep fascia (Figs. 1, 2, 3 and 4). On T1-weighted images, the lesion showed a heterogeneous high signal intensity, indicating an adipose tissue component. T2-weighted images showed a more intense heterogeneous high signal than in normal muscles but not as intense as the fluid signal seen within VM (Figs. 1, 2, 3 and 4). Less commonly, the lesion only involved the deep fascia over muscles, demonstrating a thickened fascia by heterogeneous high signal intensity. Fatty component contained high signal intensity can be seen on MRI. Lesions extended along fascial layers and could disrupted the deep fascia, demonstrating transfascial infiltration (Fig. 2). Lesions frequently surrounded the neurovascular structures within the leg. Nerves were encompassed in thick peri-neural venous anomaly, fat, and fibrosis.

Ultrasonographic studies showed a solid, heterogeneous echogenic mass entirely replacing the normal fibrillary pattern within the involved muscle (Fig. 3). Intramuscular phlebectasia were readily present on sonographic examination. There was no arterio-venous shunt or increase in the arterial flow on color Doppler interrogation.

Computed tomography (CT) showed an intramuscular mass with heterogeneous lower density than normal muscles, indicating an adipose infiltration (Fig. 3). There was hypovascularity on CT angiography (Fig. 3).

Bone changes demonstrated on radiographs included bony shortening, cortical irregularity and thinning, periosteal thickening and more extensive deformity of the bony shaft (Fig. 3). A soft tissue mass was usually indicated. Phleboliths were frequently seen in lesion (Fig. 3). Architecture of the joints was typically normal.

Venography demonstrated intralesional and extrafascial anomalous-dilated veins, and normal or prominent orthotopic veins (Fig. 3). Arteriography was unremarkable. There was no evidence of arterio-venous shunting.

### Staging and treatment

All patients were staged according to clinical features: stage I (pain stage, n = 4), stage II (contracture stage, n = 20) and stage III (deformity stage, n = 8).

All patients with stage I disease (n = 4) underwent radical resection. Patients with stage II disease (n = 20) received radical resection. Achilles lengthening was necessary in 5 patients. Four patients with lesion of gastrocnemius underwent radical resection endoscopically. In patients with stage III disease, personalized treatment approaches were required and quite variable, including radical/partial/staged resection, Achilles lengthening/tenotomy, relaxation of ankle capsule, neurolysis/neurectomy, tendon transfer, stretching exercises, and oral sirolimus/alpelisib (Table 1).

Radical resection was achieved in 28 patients whose lesions were considered resectable. One patient received 3 staged partial resections, but she was finally considered unresectable. Oral alpelisib was used postoperatively. The lesion was too extensive to resect in 3 patients who were considered unresectable. Surgery was not planned for these 3 patients. Oral sirolimus and stretching exercises were recommended to them for disease control/relief.

### Operative finding

The skin was generally normal. Operative exploration revealed significant fibrotic, fatty solid changes infiltrating the affected muscles, deep fascia and fat planes. The superficial subcutaneous fat was often uninvolved, having a lighter color. Abnormal intralesional fat usually had a darker or tan color. The abnormal fibro-adipose lesion was seen outside the deep fascia and muscles, extending along the fascia and neurovascular bundles. Affected fascia became thickened by fibro-adipose component. Veins within and around the fibro-adipose tissue were increased and dilated. Incision of affected fascia revealed unencapsulated fibro-adipose tissue infiltrating muscle, containing clustered frogspawn-like small vein lakes. Isolated strands of dilated, serpiginous veins were often seen adjacent to major neurovascular bundles (Figures 1, 2 and 3).

Dense fibro-adipose tissue was typically seen within individual muscles/groups, completely replacing the skeletal muscle along the muscular fascia. Some lesions extended across the muscular fascia to involve the adjacent muscle. In some instances, the lesion only involved deep fascia, manifesting as fascia thickening with dense fibro-adipose infiltration, and densely adhering to the underlying muscle. This dense fibrotic tissue usually could not be differentiated from that resulted from previous sclerotherapy/embolization. Some lesions also directly involved the musculotendinous junctures and tendons. Direct bony infiltration by FAVA was not observed. Bony changes on images could be interpreted as secondary reactions, or results from extrinsic compression. Occasionally, metaplastic bone tissue could be seen within the lesion, especially in elder patients (Figures 1, 2 and 3).

Abnormal adipose tissue could be seen around the neurovascular bundles. Named arteries were normal with small feeding arteries branching from normal axial arteries and their intramuscular arborizations. The accompanying veins along the named arteries usually communicated with clusters of small venous lakes and dispersed small lakes within the lesion. There was no large spongy, sinusoidal venous structure typically seen within common VM. In case of nerve involvement, the accompanying nerves often had a darker tinge or blue-black. Epineural involvement is common. Perineural and intraneural infiltration were not infrequent. Epineural and perineural lesions were dissected free when possible. Smaller sensory or noncritical nerves which were involved were sacrificed. Intraneural lesions were residuated when critical nerves were involved, which may account for post-operative neuropathic pain.

If open surgery, careful planning for skin coverage may be required. Hemostasis may be difficult to achieve. Feeding and draining vessels of the lesion should be identified and ligated. Repair of the nearby, normal larger vessels was sometimes necessitated.

When endoscopic surgery decided, careful planning for ports incision may be needed. Two or three 5 mm ports incisions were made beyond the lesion edge. In later years, endoscopic approach was preferred to resect the lesions within superficial muscles, such as gastrocnemius (Fig. 4). For deeper lesions, it was difficult to achieve radical resection through this approach, limited by the surgical workspace.

### Histopathological finding

Surgical specimen typically showed poorly demarcated fatty or fibro-fatty masses with dispersed or clustered venous nodules infiltrating skeletal muscle with frequent extension beyond the fascia. Venous nodules consisted of thin-walled, rounded, back-to-back, blood-filled sacs, intermingled with gray fibrotic tissue and yellow fat. Abnormal and irregularly small- and medium-sized veins with muscularizing were also commonly seen. Nerves were surrounded by dense fibrous tissue and infiltrated by abnormal vascular channels or exhibited perineural hyperplasia. There were also invariably lymphoplasmacytic infiltration, small foci of microcystic lymphatic malformation, metaplastic bone, and myxoid stroma, and elastosis.

### Outcome

Median follow-up duration from time of confirmed diagnosis at center was 20 months (ranged 6–32 months). Median follow-up after surgery was 21 months (ranged 6–32 months).

All patients with stage I (n = 4) or stage II disease (n = 20) underwent radical resection, obtaining compete relief of pain and retained normal or near normal range of joint motion, and improving atrophy. No recurrence was observed during follow-up.

In patients with stage III disease (n = 8), one patient continued to show loss of ankle plantar flexion, but he had improving atrophy and no pain after radical resection. One patient underwent radical resection and retained near normal range of knee motion and re-walked. One patient with staged partial resection and postoperative oral alpelisib had mild pain and some improvement of ankle motion. One patient with FAVA of extensor carpi ulnaris returned to be normal after radical resection. One patient with radical resection experienced complete relief of pain and re-walked, but she had a loss of ankle plantar flexion resulting from Achilles tenotomy. After radical resection, no recurrence was observed during follow-up. Three patients with unresectable lesion received oral sirolimus and stretching exercises, resulting in mild pain and improving atrophy, but the improvement of ankle motion was limited.

Considering potential residual disease in case of neural involvement, postoperative neuropathic pain was managed with oral sirolimus or alpelisib. The pain disappeared within days or weeks and did not recur after ceasing oral therapy during follow-up.

## Discussion

FAVA is listed as a provisionally unclassified vascular anomaly in the latest International Society for the Study of Vascular Anomalies (ISSVA) classification [11], differing from vascular malformations in its dominant solid-mass component. The fibro-adipose vascular mass can replace normal muscle tissue and involve surrounding vital neurovascular bundles. Consequently, the manifestation and management of FAVA differs from other vascular tumors or malformations. However, FAVA is commonly mistaken by practitioner as a VM or lymphatic malformation because of intramuscular involvement and local edema. There is a clear distinction among these anomalies. It is important to differentiate FAVA from common VM because the management recommendations are quite different.

A clear diagnosis of FAVA can typically be established by physical examination, ultrasonography, and MRI. FAVA patients typically present with 3 features: a solid mass, local pain, and a variable flexion contracture involving the joint. Therefore, FAVA should be suspected whenever a painful and solid mass is noted in muscle(s) of a limb. A solid, relatively noncompressible mass replacing the normal fibrillary pattern of the affected muscle can be identified on ultrasonography [1]. Within the mass, a low signal intensity on both T1- and T2-weighted images indicates a fibrotic tissue. A high signal on T1-weighted images but low signal intensity on fat-saturated T2-weighted sequences indicates an adipose component within lesion. A Low signal on T1-weighted images but high fluid signal intensity on T2-weighted sequences is indicative of venous component within the mass. These imaging features indicate a solid, fibro-adipose venous component contained intramuscular lesion. Based on all points above, it is not difficult to diagnose FAVA, so a biopsy is usually not required for diagnosis.

Recently, somatic, mosaic gain-of-function mutations of the *phosphatidylinositol-4,5-bisphosphate 3-kinase catalytic subunit alpha (PIK3CA)* gene were identified in FAVA lesion [6]. It belongs to *PIK3CA*-related overgrowth spectrum (PROS). This spectrum includes CLAPO syndrome (capillary malformation of the lower lip, lymphatic malformation of the face and neck, asymmetry and partial/generalized overgrowth), CLOVES syndrome (congenital lipomatous overgrowth, vascular malformations, epidermal nevi, scoliosis/skeletal and spinal anomalies), Klippel-Trénaunay syndrome (CLVM, combined capillary-lymphatic-venous malformation), DCMO (diffuse capillary malformation with overgrowth), FAO/HHML (fibroadipose hyperplasia or overgrowth/hemihyperplasia-multiple lipomatosis), FAVA, etc. [12]. Sequencing of *PIK3CA* can help the diagnosis and management of PROS, may playing an important role in accurate diagnosis of FAVA in some cases. Therefore, *PIK3CA* mutation detecting in specimens can be considered to aid the diagnosis of FAVA if clinical features are atypical. But, it is noteworthy that somatic and mosaic *PIK3CA* mutation is not a confirmative term for FAVA diagnosis. This mutation is not indispensable for clinical diagnosis in typical FAVA patients. Based on typical clinical symptoms, imaging demonstrations and histopathology, it is easy to diagnose for clinicians.

Correct and early diagnosis can offer the benefit of earlier surgery and avoid ineffective or potentially harmful treatment. Sclerotherapy may mildly reduce pain shortly; however, it seems ineffective for pain control in the long term [2, 5]. Additionally, sclerotherapy may aggravate the fibrotic tissue producing, result in a nerve injury, and be potentially harmful to contracture. In our patients with early stage disease (I/II), surgery alone can offer a satisfactory outcome. The radical resection is essential. Before referral, one patient had progressive loss of elbow motion after partial resection and sclerotherapy. She had a stage II disease before treatment. With earlier recognition, patients have a early stage disease at surgery, contracture and nerve involvement can be treated or prevented. With earlier surgery, cure can be expected.

In patients with stage III disease, radical resection is usually impossible. The individual management is required. Surgery remains the first choice if possible and often includes radical resection, tendon lengthening/tenotomy, neurolysis/neurectomy, capsulotomy, and tendon transfer if required. In patients with unresectable lesions, oral sirolimus or alpelisib is used for pain relief and disease control. Sirolimus therapy has a promising outcome in our patients. Oral alpelisib also successfully managed the pain in one patient. They can undergo subsequent stretching exercises when pain is resolved. Limb atrophy and contracture would be improved. Short term sirolimus or alpelisib was also used to manage postoperative neuropathic pain in case of neural involvement for potential residual disease. Amputation is not recommended in our center, because unresectable lesions associated severe pain, contracture, and atrophy can be always largely improved after sirolimus or alpelisib and physical exercises are given. Long term side-effects of oral therapy remain unknown. Since somatic and mosaic *PIK3CA* mutations have been identified in FAVA, alpelisib or sirolimus efficacy evaluation depending on *PIK3CA* genotype, cellular response, and clinical response is warranted and can be expected in future prospective studies.

All FAVA patients had deep fascia involvement. Two lesions only involved deep fascia in our patients. Some lesions had a transfascial infiltration. These findings suggest a fascia origin of FAVA. Accordingly, some authors reported the mTOR pathway components were expressed in abnormal fibrous tissue, adipose tissue and vascular anomalies of FAVA, suggesting that FAVA might be a mesenchymal origin caused by PI3K/AKT/mTOR pathway [6]. As disease progresses, abnormal tissue infiltrate into adjacent muscles and neurovascular bundles.

Given the impression of non-congenital origin of FAVA, in contrast to other related malformations or other diseases within PROS, we payed attention to onset causes during disease history collection. We felt a distinct pattern of trauma might be a possible initial factor, but previous trauma was only noticed in two patients. In children, previous various traumatism may be forgotten or not noticed, so they could not provide a clear history of trauma. In our impression, the traumatism or injury from sclerotherpy seems to be the accelerator for disease progression.

Four patients underwent endoscopically radical resection. This is a novel surgical technique. We believe that providing details regarding this new approach would be beyond the scope of this presentation and has been reported in a separate paper [13].

Follow-up duration in this cohort is limited, so pain and disease recurrence must require constant vigilance. However, we think the rarity and complexity of this entity, the paucity of reports, and the heterogeneity of treatment modalities mean our patients still provide valuable references for clinicians. Our staging system, management principle, and detailed imaging description for diagnosis also offered additional information to existing literature. There was also a lack of formal patient-reported outcome measure in this study. The indication for surgery, the resectability deciding, and the extent of resection is not uniform in literature and this paper. We did not have a comparison between our patients and patients in current literature. However, these are the general natures of management for rare diseases with evolving recognition and experience.

## Conclusion

FAVA is a rare vascular anomaly that featured by local mass, severe pain, and joint contractures in a limb. It is female predominant, usually presented in childhood. Earlier awareness of this disease can reduce misdiagnoses. Surgery-based comprehensive management can typically improve pain and contracture. Oral sirolimus or alpelisib plays an important role in treatment of unresectable lesions and major nerve involvement. Surgery alone can be curative in early stage FAVA. Whether the disease would recur remains unknown because of a lack of long-term, prospective studies, especially in patients with potential residual disease in major nerve, although we have not yet observed local recurrence.

## Data Availability

No datasets were generated or analysed during the current study.

## Abbreviations

FAVA: Fibro-adipose vascular anomaly
ISSVA: International Society for the Study of Vascular Anomalies
CT: Computed tomography
MRI: Magnetic resonance imaging
VM: Venous malformation
*PIK3CA*: Phosphatidylinositol-4,5-bisphosphate 3-kinase catalytic subunit alpha
PROS: *PIK3CA*-related overgrowth spectrum
CLAPO syndrome: Capillary malformation of the lower lip, lymphatic malformation of the face and neck, asymmetry and partial/generalized overgrowth
CLOVES syndrome: Congenital lipomatous overgrowth, vascular malformations, epidermal nevi, scoliosis/skeletal and spinal anomalies
Klippel-Trénaunay syndrome: CLVM, combined capillary-lymphatic-venous malformation
DCMO: Diffuse capillary malformation with overgrowth
FAO/HHML: Fibroadipose hyperplasia or overgrowth/hemihyperplasia-multiple lipomatosis
PI3K: Phosphatidylinositol-3-kinase
mTOR: mammalian target of rapamycin
